# Polyethylenimine Assisted Bio-Inspired Surface Functionalization of Hexagonal Boron Nitride for Enhancing the Crystallization and the Properties of Poly(Arylene Ether Nitrile)

**DOI:** 10.3390/nano9050760

**Published:** 2019-05-17

**Authors:** Yajie Wang, Lifen Tong, Yong You, Ling Tu, Meirong Zhou, Xiaobo Liu

**Affiliations:** Research Branch of Advanced Functional Materials, School of Materials and Energy, University of Electronic Science and Technology of China, Chengdu 611731, China; wangyajie0817@126.com (Y.W.); yourkeaib@163.com (Y.Y.); TLuestc@163.com (L.T.); zhoumeirong94@163.com (M.Z.)

**Keywords:** poly(aryl ether nitrile), crystallization behavior, hexagonal boron nitride, Polydopamine, polyethylenimine

## Abstract

Semi-crystalline poly(arylene ether nitrile) (PEN) has exhibited remarkable potential in various fields. However, the inherent drawbacks of PEN such as slow crystalline rate and low crystallinity limit its further development. To alleviate this problem, the choice of nanofiller as nucleation agent and the interfacial compatibility between nanofiller and PEN matrix are two momentous factors that need to be considered. Accordingly, in this work, functionalized hexagonal boron nitride (h-BN@(PDA+PEI)) was successfully synthesized via polyethylenimine (PEI) assisted bio-inspired surface functionalization, and then homogeneously dispersed in the PEN resin using solution casting method to obtain functional polymer nanocomposite films with strengthening the crystallization behavior, mechanical and dielectric properties. Various testing methods including differential scanning calorimetry (DSC), scanning electron microscopy (SEM), X-ray diffraction (XRD), and polarizing microscope (POM) were applied to intricately analyze the effect of h-BN@(PDA+PEI) on the crystallization behavior of PEN composites. The testing results certificated that the h-BN@(PDA+PEI) can effectively improve the crystallinity (from 6.56% to 14.90%), and the spherulite size of PEN was reduced while the nucleation density of nanocomposites was raised. Furthermore, the non-isothermal crystallization kinetics demonstrated that 2 wt% h-BN@(PDA+PEI) could significantly reduce the cold crystallization temperature (*T_p_*) and the crystallization activation energy (*E_a_*) (from 359.7 KJ/mol to 292.8 KJ/mol), while it improved the crystallization rate (*K_c_*) of PEN. In addition, the mechanical and dielectric properties of nanocomposite films were also reinforced to further broaden the application of semi-crystalline PEN. Therefore, the h-BN@(PDA+PEI) can function as an effectual nucleating agent and enhance the performance of PEN.

## 1. Introduction

Semi-crystalline polymers play an important role in industrial practical applications, whose physical properties depend largely on their crystallization behavior. With the ever-increasing development of semi-crystalline engineering plastics, the crystallization behavior of them has attracted extensive attention. As a novel special engineering polymer, semi-crystalline poly(arylene ether nitrile) (PEN) has aroused many attentions due to its prominent chemical and physical properties, such as brilliant mechanical performance (100–120 MPa), excellent radiation resistance and high thermal stability (*T_5%_* > 480 °C), which allow it to show off various applications in automotive, military and aerospace fields [1,2,3,4]. However, the semi-crystalline PEN containing rigid groups possesses slow crystalline rate, low crystallinity, and inherent brittleness, which limits its further practical application [5,6]. The incorporation of some inorganic nanofillers into the polymer matrix is a traditional strategy to improve the crystallization ability and the mechanical properties [7,8,9,10,11], including metal oxides [12], silica [13], talc [14], graphene [15], carbon nanotubes [16] and so on. Unfortunately, there are currently few studies concentrated on the effect of nanoscale fillers on the crystallization behavior of semi-crystalline PEN. Up to now, there has been only Yang et al. who reported the effect of the aluminum oxide (Al_2_O_3_) and multi-walled carbon nanotubes (MWCNTs) on the crystallization behavior of PEN to the best of our knowledge [17]. However, the corresponding mechanism analysis is not exhaustive. In addition, the interfacial compatibility between the nanofiller and the polymer is also a significant factor to be considered, or else most inorganic nanofillers will undergo obvious aggregation in the polymer matrix due to the poor interfacial interaction, which will cause the deterioration of nucleation performance and mechanical properties of nanocomposites.

More recently, hexagonal boron nitride (h-BN) with structure similar to graphene exhibits diverse applications due to its excellent thermal stability, high mechanical properties, outstanding thermal conductivity and superb oxidation resistance [18,19]. Compared with other nano-nucleation agents like MWCNTs and graphene, h-BN is environmentally benign and cheap. Moreover, h-BN with the large nucleation surface can provide more nucleation sites for polymer crystallization, which has been applied as an excellent nucleating agent for polymer matrix [20,21]. In view of this, the practical applications of PEN composites will be broadened by introducing h-BN into the semi-crystalline PEN matrix to improve its crystallization.

On the other hand, the well dispersibility of nanofiller in the polymer matrix is beneficial for improving the nucleation capability of h-BN and the physical properties of the composites. It was found that the surface modification is an effective method to solve this problem. Recently, polydopamine (PDA) coating, which stems from the self-polymerization of dopamine (DA), as a green and easy approach of surface modification due to no dangerous solvent involved and moderate reaction temperature, has represented multiple applications in many fields, such as water purification [22], batteries [23] and catalysis [24], etc. However, this process is normally time-consuming, which leads to the PDA layer heterogeneous and impact. It has been reported that low molecular weight polyethyleneimine (PEI) could accelerate the DA polymerization process and was beneficial for the formation of compact and homogeneous polymer (PDA-PEI) layer via Schiff-base or Michael addition reaction [25]. In addition, the resulting wrapping layer endows the inorganic nanofiller with abundant polar groups, which could enhance the dispersibility of inorganic nanofiller in the polymer matrix through polar interaction.

In consideration of the discussions mentioned above, in the present work, novel h-BN@(PDA+PEI)/PEN nanocomposites were designed. The h-BN was firstly treated through the method of PEI assisted bio-inspired surface functionalization. PDA and its derivatives formed through the oxidation and self-polymerization of DA, followed by further reaction with PEI on the surface of h-BN. Thus, owing to the abundant polar groups endowed by PDA and PEI, the improvement of the dispersibility of h-BN could be realized by solution casting process. The as-prepared h-BN@(PDA+PEI)/PEN nanocomposite films displayed enhanced crystallization ability, mechanical properties and dielectric constant, which can be potentially used in practical industrial applications.

## 2. Experimental

### 2.1. Materials

Potassium carbonate (K_2_CO_3_), 2,6-Dichlorobenzonitrile (DCBN), resorcinol (RS), hydroquinone (HQ), *N*-methyl-2-pyrrolidone (NMP) and toluene were purchased from Chengdu Changzheng Chemicals, Chengdu, China. Dopamine hydrochloride (DA), branched poly(ethylene imine) (PEI; Mw = 600), tris (hydroxymethyl) aminomethane (Tris-HCl) and hexagonal boron nitride (h-BN) were obtained from Aladdin, Chengdu, China.

### 2.2. Synthesis of PEN

**Figure 1:**
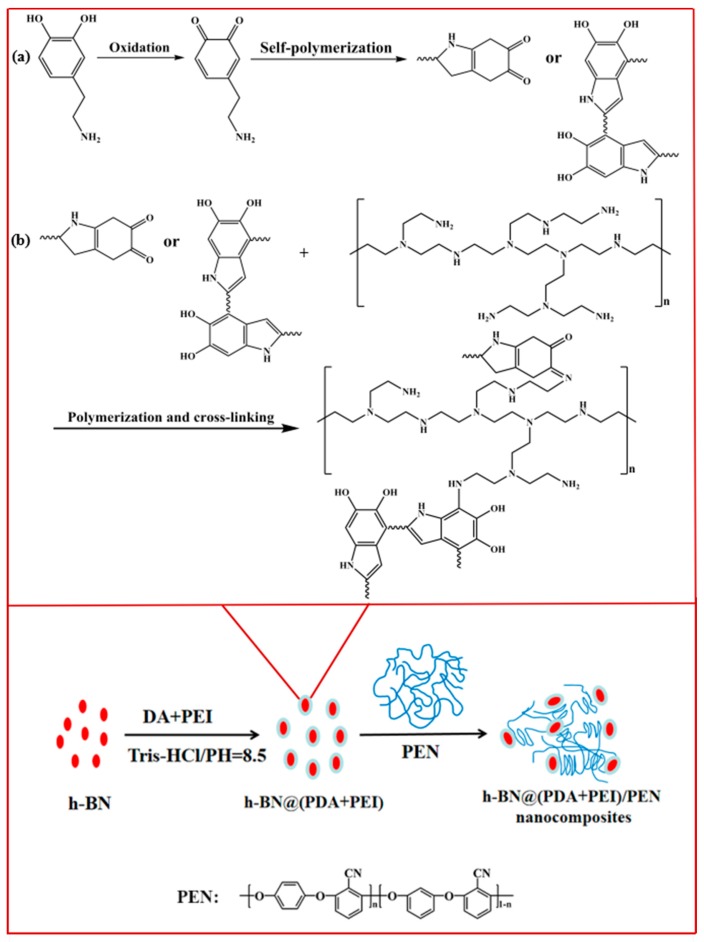
Preparation process of functionalized h-BN/PEN nanocomposite films and possible chemical reaction between PDA and PEI.

The typical semi-crystalline PEN (hydroquinone/resorcinol = 8/2 by mol) was synthesized according to our previous work [26] and its structure was displayed in Figure 1. The inherent viscosity of it was 0.73 dL/g in the *N*-methyl-2-pyrrolidone (NMP) at 75 ± 0.1 °C.

### 2.3. PEI Assisted Bio-Inspired Surface Functionalization of h-BN

200 mg of h-BN powders were dispersed in Tris-HCl buffer solution (50 mL, 600 mg, pH = 8.5) by using the ultrasonic bath. Then 100 mg of DA and 300 mg of PEI were added in the above solution under strongly mechanical stirring at 25 °C for 8 h. Finally, the as-prepared functionalized h-BN powders were centrifuged, washed and dried at 60 °C in vacuum overnight and named as h-BN@(PDA+PEI).

### 2.4. Preparation of h-BN@(PDA+PEI)/PEN Nanocomposite Films

Functionalized h-BN/PEN composite films with different h-BN@(PDA+PEI) contents (0, 0.5, 1, 2 and 3 wt%) were fabricated by a simple solution casting method. Firstly, h-BN@(PDA+PEI) was evenly dispersed in NMP by sonication for 30 min, and then modified h-BN was added into the PEN solution with mechanical agitation at 120 °C for 1 h. Finally, the mixture was cast onto a smooth and clean glass plate before heating at 80 °C, 100 °C, 120 °C, 160 °C, 200 °C each for 2 h, respectively.

### 2.5. Characterization

The cold crystallization behavior of h-BN@(PDA+PEI)/PEN composite films was investigated on DSC-Q100 (TA Instruments, New Castle, DE, USA). Firstly, the samples were heating to 370 °C for 10 min to eliminate the thermal history. Afterwards, the films were cooled from 370 °C to 50 °C with a rate of 60 °C/min rapidly. Finally, the samples were heated again with a rate of 5, 10, 15, and 20 °C/min from 50 °C to 370 °C, respectively. The crystal structure of h-BN and the crystallinity of h-BN@(PDA+PEI)/PEN composites were investigated by the XRD (RINT2400, Rigaku, Japan). The crystal morphologies of neat PEN and h-BN@(PDA+PEI)/PEN nanocomposites were performed by SEM (JSM, 6490LV, Tokyo, Japan) and POM (MP41, Beijing, China), respectively. XPS (ESCALAB250, Waltham, MA, USA) and FT-IR (Shimadzu 8400S, Kyoto, Japan) were used to inspect the functionalization of h-BN. TEM (JEM-2100F, JEOL, Tokyo, Japan) was carried out to investigate the micromorphology of modified h-BN. The dielectric properties of nanocomposites were conducted by Tong Hui TH 2819A precision LCR meter (Shanghai, China). Mechanical properties were investigated by using universal testing machine (SANS, CMT6104, Shanghai, China).

## 3. Results and Discussion

### 3.1. Structure and Morphology of Pure h-BN and h-BN@(PDA+PEI)

**Figure 2:**
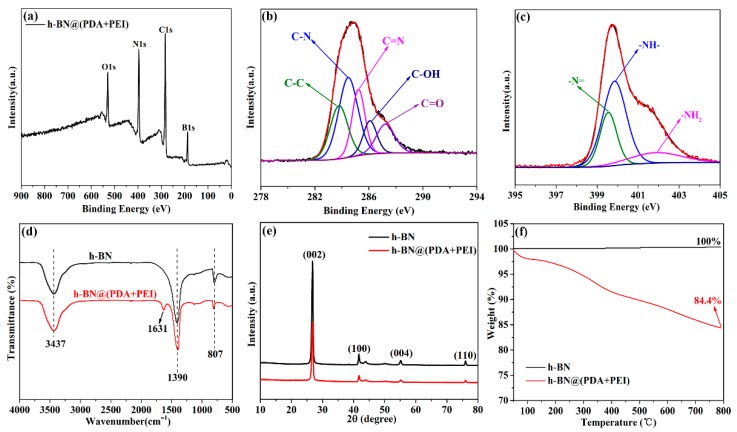
The characterization of h-BN samples: (**a**) XPS wide scan; (**b**) C 1s spectra; (**c**) N 1s spectra; (**d**) FT-IR spectra; (**e**) XRD patterns; (**f**) TGA curves.

The surface chemical composition of h-BN@(PDA+PEI) is verified by XPS and the results are shown in Figure 2a–c. As depicted in Figure 2a, the h-BN@(PDA+PEI) is composed of B, N, C and O elements. The B 1s (188.2 eV) and N 1s (399.9 eV) peak demonstrate the presence of h-BN. To further validate the existence of PDA-PEI, the C 1s and N 1s spectra of h-BN@(PDA+PEI) are given in Figure 2b,c, respectively. The C 1s spectra can be curve-fitted with five peaks at the binding energy of 283.8 eV, 284.5 eV, 285.2 eV, 286.2 eV and 287.4 eV, corresponding to C–C, C–N, C=N, C–OH and C=O, respectively. The C–N stems from PDA or PEI, and the existence of C=N explains the possible chemical reaction between PEI and PDA [27]. In addition, three species of N1s peak assigning to –N= (399.6 eV), –N–H– (399.8 eV), and –NH_2_ (401.9 eV) are shown in Figure 2c. These results are consistent with the reaction mechanism illustrated in Figure 1. Furthermore, the FT-IR spectra of pure h-BN and h-BN@(PDA+PEI) are presented in Figure 2d. The adsorption peaks at 807 cm^−1^ and 1390 cm^−1^ can be observed in both of samples, which are ascribed to the bending and stretching vibration of B–N, respectively. However, after surface functionalization by PDA and PEI, a new characteristic peak at 1631 cm^−1^ of C=N emerges in h-BN@(PDA+PEI), which is in accordance with the phenomenon mentioned above. Therefore, these results verify that the h-BN has been modified by PDA and PEI successfully.

The structure of pure h-BN and h-BN@(PDA+PEI) are detected by XRD and displayed in Figure 2e. The diffraction peaks at 26.7°, 41.6°, 55.1° and 75.9° belong to (002), (100), (004) and (110) planes [28], respectively. And there is no diffraction peaks for impurities, demonstrating that the sample is highly pure. Moreover, the same diffraction peaks are also represented in the h-BN@(PDA+PEI), the intensity of which are subdued obviously. Nonetheless, the positions of diffraction peaks for h-BN@(PDA+PEI) are in agreement with those of pure h-BN, recommending that the surface functionalization do not make the crystalline phase of h-BN change.

The thermal degradation behavior of pure h-BN and h-BN@(PDA+PEI) is shown in Figure 2f. It can be found that the weight percentage of pure h-BN still remains invariable even though the temperature rises to 800 °C, which proves the excellent thermal stability of pure h-BN. By contrast, in the TGA curve of h-BN@(PDA+PEI), a weight loss from room temperature to 800 °C can be observed due to the decomposition of PDA and PEI, which is approximately 15.6 wt% [29].

**Figure 3:**
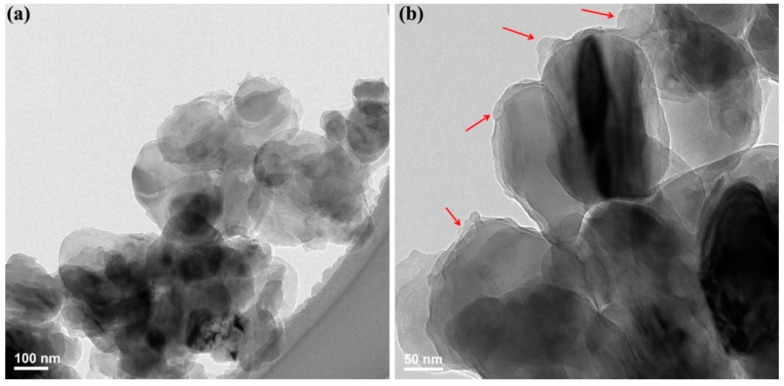
TEM images of (**a**) and (**b**) h-BN@(PDA+PEI).

The micro-structure of h-BN@(PDA+PEI) is confirmed by TEM and shown in Figure 3. It can be found that the h-BN presents a typical thin lamellar structure with a lateral dimension of 100–300 nm. The surface of h-BN@(PDA+PEI) is obscure and coated by a thin layer of irregular substance (marked with red arrows) after co-modification by PDA and PEI. Therefore, combined with XPS, FT-IR spectra, XRD patterns, TGA curves and TEM images, it can be concluded that the PDA-PEI layer was successfully wrapped on the surface of h-BN.

### 3.2. The Effect of h-BN@(PDA+PEI) on the Non-Isothermal Crystallization of PEN

**Figure 4:**
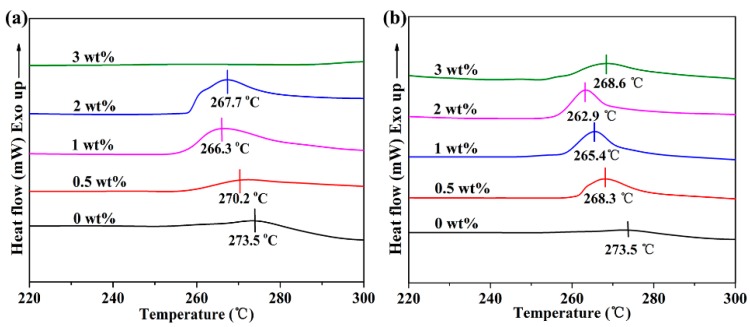
The effect of h-BN and h-BN@(PDA+PEI) loading on the cold crystallization of nanocomposites: (**a**) h-BN/PEN nanocomposites; (**b**) h-BN@(PDA+PEI)/PEN nanocomposites at the heating rate of 10 °C/min, respectively.

The nucleation performance of pure h-BN and modified h-BN is detected by DSC non-isothermal cold crystallization curves, respectively. As seen in Figure 4, both h-BN@(PDA+PEI) and pure h-BN can promote the cold crystallization of PEN to some extent. The cold crystallization temperature (*T_p_*) of h-BN/PEN nanocomposite with 1 wt% h-BN decreases from 273.5 °C to 266.3 °C in comparison with pure PEN. However, the cold crystallization temperature (*T_p_*) of h-BN@(PDA+PEI)/PEN nanocomposite occurs at a lower temperature of 262.9 °C with a small amount of h-BN@(PDA+PEI) (2 wt%) and cold crystallization peak is sharper, demonstrating that the crystallization arises more quickly with excellent crystal perfection and the narrow allocation of crystal size, which shows that h-BN@(PDA+PEI) has better heterogeneous nucleation effect than h-BN. The excellent nucleation effect of h-BN@(PDA+PEI) can be described as follows. On one hand, h-BN with high specific surface area can provide more nucleation sites for PEN crystallization. On the other hand, a large number of polar groups (–OH and –NH_2_) endowed by PDA and PEI can further enhance the interfacial compatibility between PEN matrix and h-BN filler [20]. The two aspects promote the cold crystallization of PEN. In addition, the cold crystallization of PEN can be obstructed by h-BN with high loading (3 wt%). This can be explained that more free volume is occupied due to the high content of nanofillers, which limits the movement of the polymer chains to some extent.

To further examine the cold crystallization behavior of various h-BN@(PDA+PEI)/PEN nanocomposites, the non-isothermal crystallization kinetics of nanocomposite films were researched [30,31,32,33]. The Avrami equation was employed as follows.

(1) $$1-X_{t}=\exp(-K_{t}t^{n})$$

Equation (1) is converted to as below by taking double logarithms.

(2) $$\ln\left[-\ln(1-X_{t})\right]=\ln K_{t}+n\mathrm{lnt}$$

t means the crystallization time; *K_t_* is the crystallization rate constant, depending on nucleation and growth parts; n represents the Avrami constant relating to the nucleation mechanism and crystal growth geometry; Thence, according to the Equation (2), the *K_t_* and n could be acquired from the intercept and the slope, respectively. Furthermore, the *K_t_* can be modified by the Equation (3), β represents the cooling rate or heating rate.

(3) $$\ln K_{c}=\frac{\ln K_{t}}{\beta }$$

*X_t_* represents the relative crystallinity and could be defined as Equation (4) [34]. *T_0_* and *T_∞_* are the starting and ending crystallization temperatures, respectively.

(4) $$X_{t}=\frac{\int_{T_{0}}^{T}\frac{\partial H_{c}}{\partial T}dt}{\int_{T_{0}}^{T_{\infty }}\frac{\partial H_{c}}{\partial T}dt}$$

**Figure 5:**
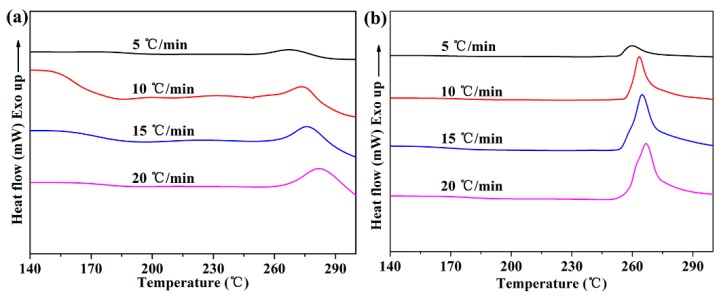
The effects of the heating rate on the cold crystallization temperature of neat PEN (**a**) and PEN nanocomposite with 2 wt% h-BN@(PDA+PEI) (**b**).

**Figure 6:**
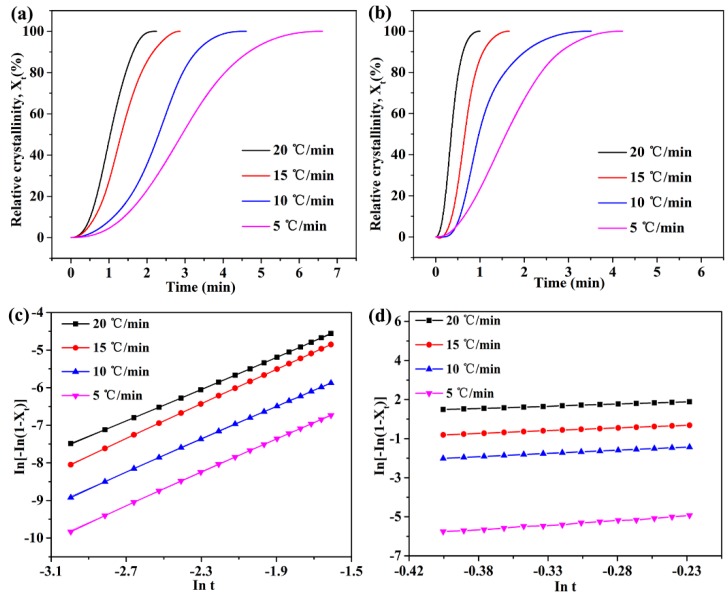
The relative crystallinity and Avrami of neat PEN (**a**,**c**) and PEN nanocomposite film with 2 wt% h-BN@(PDA+PEI) (**b**,**d**), respectively.

Figure 5 demonstrates the DSC curves of neat PEN and functionalized h-BN/PEN nanocomposite with 2 wt% h-BN@(PDA+PEI) at various heating rates and the cold crystallization temperatures (*T_p_*) of PEN nanocomposites are summarized in Table 1 in detail. It could be discovered that all the DSC curves have a cold crystallization peak and the range of the cold crystallization temperature (*T_p_*) is 258.5 °C to 278.9 °C. The same phenomenon occurs in two DSC curves that the *T_p_* moves to the high temperature and cold crystallization peak becomes larger with the heating rate increasing, which is mainly because the PEN crystals have the less nucleation and crystal growth time as the heating rate increases [35]. At the same time, the PEN nanocomposite shows the sharper cold crystallization peak and lower cold crystallization temperature with 2 wt% h-BN@(PDA+PEI) at each heating rate. The relative crystallinity (*X_t_*) is an important parameter in the study of crystallization kinetics. Figure 6 displays the typical relative crystallinity versus the crystallization time for neat PEN (Figure 6a) and PEN nanocomposite with 2 wt% h-BN@(PDA+PEI) (Figure 6b). The normal S-shape is exhibited in two experimental curves, which is well consistent with the nucleation and crystal growth process of PEN. Moreover, it is clear that the crystallization time is significantly shortened with the increasing of heating rate. Simultaneously, it can be found that the addition of modified h-BN significantly reduces the crystallization time of PEN.

Figure 6c,d display the curves of the ln[−ln(1−*X_t_*)] versus lnt of neat PEN and PEN nanocomposite with 2 wt% h-BN@(PDA+PEI) at various heating rates, respectively. ln*K_t_* and exponent n were successfully obtained by calculating the intercept and slope of lines. The detailed data are listed in Table 1. It could be observed that the exponent n value of neat PEN is about 2 because of the higher nucleation energy barrier for pure PEN, so only a small part of spherulitic growth occurs in the primary nucleation process, and the growth is mainly two-dimensional [36]. However, exponent n increases with the loading of h-BN@(PDA+PEI) increasing (no more than 2 wt%) at each heating rate. For instance, exponent n clearly increases from 2.23 to 3.31 as h-BN@(PDA+PEI) content increasing from 0 wt% to 2 wt% when the heating rate is 5 °C/min. This demonstrates a normal three-dimensional growth of PEN nanocomposites and indicates the excellent heterogeneous nucleation ability of h-BN@(PDA+PEI) once again. Moreover, the *K_c_* increases with the loading of h-BN@(PDA+PEI) increasing (no more than 2 wt%) under the same heating rate. Normally, a higher *K_c_* value means that crystallization rate of the matrix is faster at the same heating rate [37]. It indicated that the crystallization rate of PEN matrix is improved and the nucleation method of PEN is changed by the introduction of modified h-BN.

**Figure 7:**
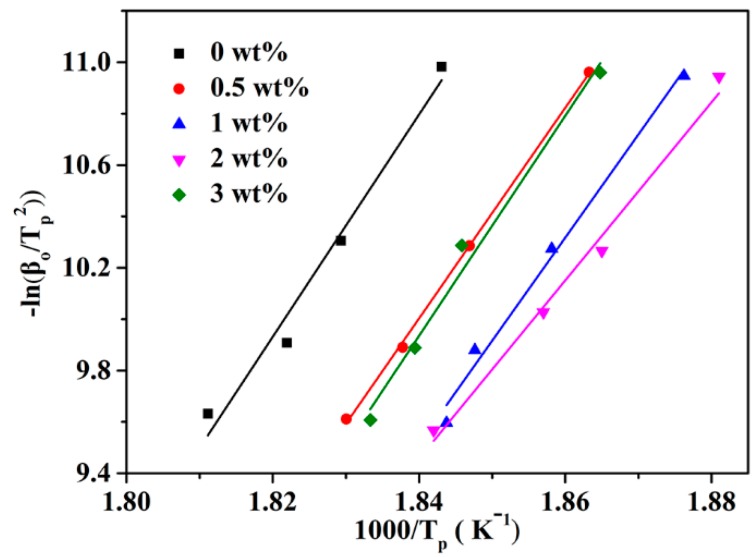
The activation energy of crystallization of various h-BN@(PDA+PEI) nanocomposites.

Furthermore, the activation energy (*E_α_*) of the crystallization of polymers could be calculated by the Kissinger method which has also been previously employed [38]. From Equation (5), the *E_α_* could be calculated from the slope of Figure 7 and the detailed data are listed in Table 1. It can be discovered that the *E_α_* of nanocomposites effectively reduces from 359.66 KJ/mol to 292.84 KJ/mol as h-BN@(PDA+PEI) contents increasing from 0 wt% to 2 wt%, which is due to the fact that the h-BN@(PDA+PEI) acts as a foreign phase for pure PEN, and the fold surface free energy and nucleation activation energy of the PEN crystal can be reduced, making the PEN crystallize easier [8].

(5) $$\ln\left(\frac{\beta }{T_{p}^{2}}\right)=\mathrm{Const}-\left(\frac{E_{a}}{RT_{p}}\right)$$

### 3.3. The Effect of h-BN@(PDA+PEI) on the Crystal Structure and Spherulite Morphology of PEN

**Figure 8:**
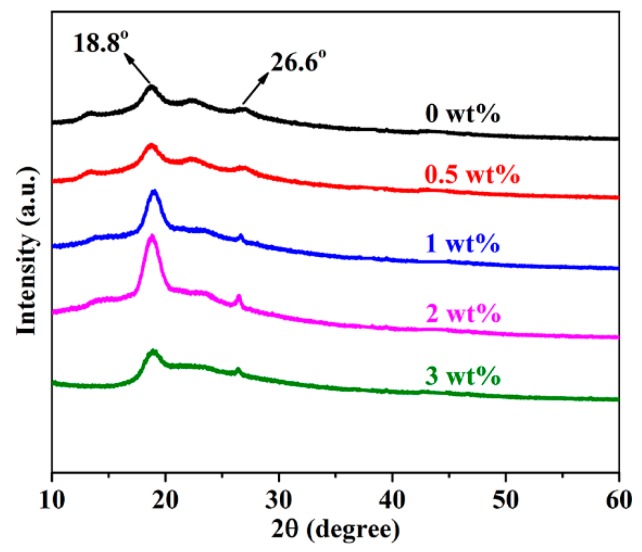
WAXD patterns of various h-BN@(PDA+PEI)/PEN nanocomposite films.

The crystallinity and crystal structure of various h-BN@(PDA+PEI)/PEN nanocomposite films have been studied by WAXD, which is displayed in Figure 8. The detailed data are showed in Table 2. It can be noticed that the diffraction peaks in all the h-BN@(PDA+PEI)/PEN composite films are all the same, demonstrating that the addition of modified h-BN does not change the crystal structure of PEN. At the same time, the diffraction peaks at 2θ = 18.8°, 26.6° become strong firstly and then weak when the functionalized h-BN contents increase. As the modified h-BN contents increase from 0 wt% to 2 wt%, the crystallinity of nanocomposites which is calculated through peak-differentiating and imitating increases from 6.56% to 14.90%. However, when the nanoparticles content is increased to 3 wt%, the movement of molecular segment is obstructed by nanofiller, which lead to the decreasing of crystallinity to some extent. This result is consistent with the DSC analysis result, indicating that functionalized h-BN could obviously promote the crystallinity of PEN.

**Figure 9:**
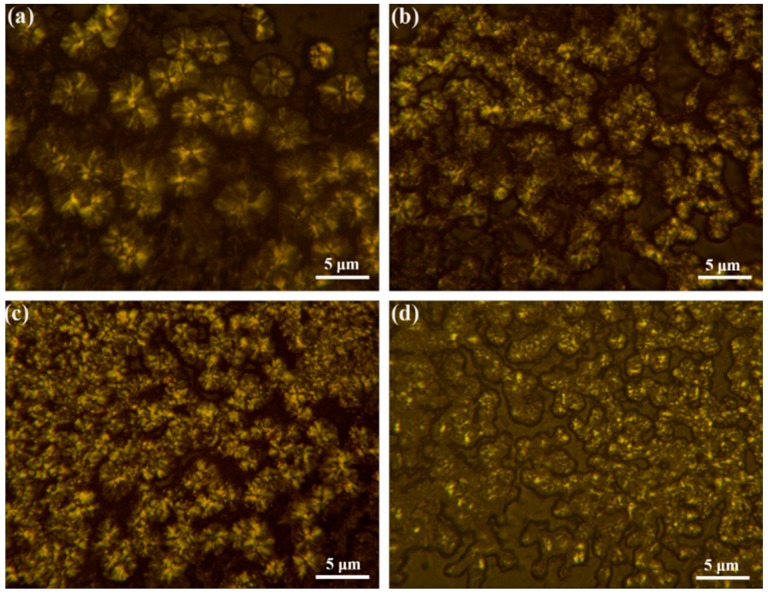
Polaring optical microscopy (POM) of the nanocomposites with different h-BN@(PDA+PEI) contents: (**a**) 0 wt%; (**b**) 0.5 wt%; (**c**) 1 wt%; (**d**) 2 wt% isothermal crystallization at 260 °C for 2 h, respectively.

**Figure 10:**
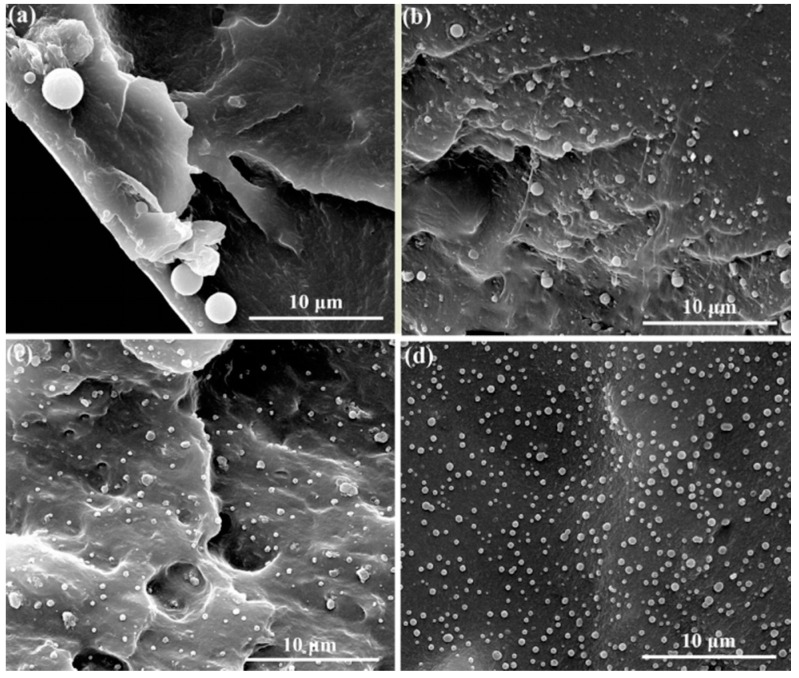
SEM micrographs of the cross-section of the nanocomposites with different h-BN@(PDA+PEI) contents: (**a**) 0 wt%; (**b**) 0.5 wt%; (**c**) 1 wt%; (**d**) 2 wt% isothermal crystallization at 260 °C for 2 h, respectively.

In order to explore the change of crystal size after the addition of modified h-BN, the POM images and SEM micrographs of the PEN nanocomposites crystallized isothermally at 260 °C for 2 h have been further investigated. Figure 9 displays the POM images of the nanocomposites, it is observed that the average spherulite diameter reaches 2.5 μm for pure PEN (Figure 9a). However, the amount of nuclei increases tremendously and more small spherulites are formed for the nanocomposites with various h-BN@(PDA+PEI) contents. Notably, the higher spherulite density and smaller crystal size of the h-BN@(PDA+PEI)/PEN nanocomposites (2 wt% h-BN@(PDA+PEI)) recommend that highly dispersed h-BN act as effective nucleation sites and promote the growth of PEN spherulites (Figure 9d). This phenomenon verifies intuitively that the heterogeneous primary nucleating ability of PEN is greatly improved by the h-BN@(PDA+PEI). Figure 10 shows SEM micrographs of PEN matrix with different modified h-BN contents, and the SEM micrographs have the same phenomenon as observed by POM. As the h-BN@(PDA+PEI) contents increase from 0 wt% to 2 wt%, the average spherulite size of the PEN decreases from about 2.5 μm to 0.5 μm and the nucleation density of PEN increases sharply. The superb nucleating effect of h-BN@(PDA+PEI) could be described as follows. The free energy barrier of PEN matrix in primary nucleation process is reduced due to the large nucleation surface of nanofiller. And then the PDA-PEI layer with a large number of polar groups can provide hydrogen bonding interactions with PEN matrix. Combined with the previous DSC curves, XRD patterns, POM photos and SEM micrographs, it suggests that h-BN@(PDA+PEI) can be developed as a highly superior nucleating agent for PEN.

### 3.4. Mechanical and Dielectric Properties of h-BN@(PDA+PEI)/PEN Nanocomposite Films

**Figure 11:**
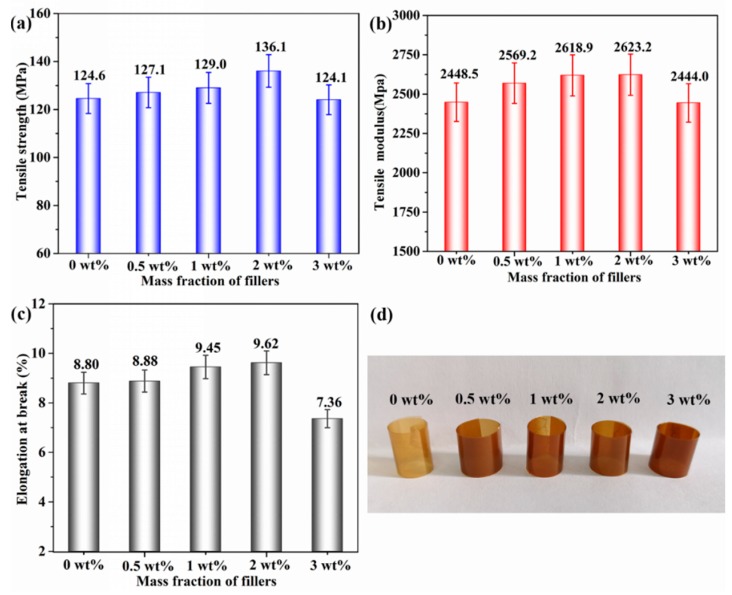
Tensile properties of h-BN@(PDA+PEI)/PEN: (**a**) tensile strength; (**b**) tensile modulus; (**c**) elongation at break and (**d**) photos of curled PEN nanocomposite films with various h-BN@(PDA+PEI) contents.

To further explore the influence of functionalized h-BN on the properties of PEN composite films, the mechanical properties of them are investigated and shown in Figure 11. As depicted in Figure 11a,b, the same fluctuation trend can be observed in both the tensile strength (T_S_) and modulus (T_M_), which increase significantly at low mass fraction of functionalized h-BN and reach a maximum value of 136.1 MPa and 2623.2 MPa with 2 wt%, respectively, indicating the increment of 9% and 7% compared with pure PEN film (124.6 MPa and 2448.5 MPa). The improvements of T_S_ and T_M_ are ascribed to the even dispersion of functionalized h-BN in the PEN matrix through strong polar interaction. However, the elongation at break (E_b_) of the nanocomposites gradually increases and then slightly decreases with the increasing modified h-BN contents. Especially, the elongation at break increases from 8.80% to 9.62% when the loading content is 2 wt% (Figure 11c), demonstrating that a small amount of h-BN@(PDA+PEI) with better dispersion in the matrix can make optional and abundant interfacial interactions between the nanofillers and PEN matrix. Moreover, the formation of small and dense spherulites reduces the voids inside the spherulites and the defects of the crystallization interface. Namely, a small amount of addition does not lead to the brittleness increase of PEN but a slight increase in toughness. As shown in Figure 11d, these films could be lightly curled into multilayer cylinders with color changes from light brown to deep brown, recommending the high flexibility of them.

**Figure 12:**
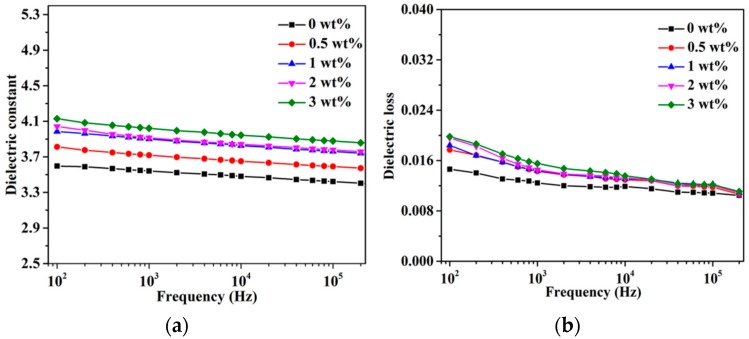
Frequency dependences of (**a**) dielectric constant and (**b**) dielectric loss of the nanocomposites films with various h-BN@(PDA+PEI) contents.

The dielectric properties of the h-BN@(PDA+PEI)/PEN nanocomposite films with different h-BN@(PDA+PEI) contents are also investigated from 1 × 10^2^ Hz to 2 × 10^5^ Hz, which is shown in Figure 12. It can be discovered that the dielectric constant and dielectric loss of all composite films slightly decrease with the frequency increasing, which shows the dependence of both of them on frequency to some extent. The dielectric constant of the nanocomposites increases from 3.5 to 4.0 as the h-BN@(PDA+PEI) increases from 0 wt% to 3 wt% at 1 kHz. Furthermore, it also can be found from Figure 12b all the films show low dielectric loss of less than 0.02, which is attributed to the PDA-PEI layer could provide a mass of polar groups to intensify the interfacial compatibility between h-BN and PEN matrix.

## 4. Conclusions

In summary, novel functional h-BN/PEN nanocomposite films were developed via a solution casting method. The results obtained in this work demonstrated that the successful PEI assisted bio-inspired surface functionalization to improve the interfacial adhesion of h-BN, leading to the enhancement of crystallization ability, mechanical properties and dielectric properties. Notably, 2 wt% h-BN@(PDA+PEI) could significantly reduce crystallization activation energy (*E_a_*) (from 359.66 KJ/mol to 292.84 KJ/mol) and the cold crystallization temperature (*T_p_*), while effectively improving the crystallinity (from 6.56% to 14.90%) and the crystallization rate (*K_c_*) of PEN composites. Moreover, the spherulite size of PEN was reduced and the nucleation density was raised by the introduction of h-BN@(PDA+PEI). Therefore, it draws a conclusion that h-BN@(PDA+PEI) can be used as a valid nucleating agent and strengthen the performance of PEN.

## Figures and Tables

**Table 1:** Detailed data of non-isothermal crystallization kinetic parameters of various h-BN@(PDA+PEI) nanocomposites.

Samples	β	n	In*K_t_*	In*K_c_*	*K_c_*	*T_p_* (°C)	*E_α_* (KJ/mol)
0 wt%	5	2.23	−3.140	−0.628	0.534	269.4	359.66
	10	2.18	−2.346	−0.235	0.791	273.5	
	15	2.29	−2.295	−0.153	0.858	275.7	
	20	2.22	−1.560	−0.078	0.925	278.9	
0.5 wt%	5	2.38	−2.582	−0.516	0.597	263.5	340.81
	10	2.27	−1.155	−0.116	0.890	268.3	
	15	2.42	−0.616	−0.0410	0.960	270.9	
	20	2.23	0.015	0.001	1.000	273.3	
1 wt%	5	2.61	−2.008	−0.402	0.669	259.8	333.93
	10	2.57	−1.156	−0.116	0.891	265.0	
	15	2.56	−0.183	−0.012	0.988	268.1	
	20	2.25	1.499	0.075	1.078	269.2	
2 wt%	5	3.31	−1.363	−0.273	0.761	258.5	292.84
	10	3.15	−0.518	−0.052	0.949	262.9	
	15	2.62	1.019	0.068	1.070	264.3	
	20	2.48	2.073	0.124	1.132	266.2	
3 wt%	5	2.15	−1.829	−0.366	0.694	263.1	355.98
	10	2.34	−0.917	−0.092	0.912	268.6	
	15	2.21	−0.136	−0.009	0.990	270.5	
	20	2.06	0.336	0.017	1.017	272.3	

**Table 2:** The crystallinity of PEN nanocomposites with various h-BN@(PDA+PEI) contents.

Sample	Neat	0.5 wt%	1 wt%	2 wt%	3 wt%
Crystallinity (%)	6.56	8.22	11.32	14.90	7.14
